# KRAS K104 modification affects the KRAS^G12D^-GEF interaction and mediates cell growth and motility

**DOI:** 10.1038/s41598-020-74463-5

**Published:** 2020-10-15

**Authors:** Chih-Chieh Chen, Chia-Yi Hsu, Hsiao-Yun Lin, Hong-Qi Zeng, Kuang-Hung Cheng, Chia-Wei Wu, Eing-Mei Tsai, Tsung-Hua Hsieh

**Affiliations:** 1 Institute of Medical Science and Technology, National Sun Yat-sen University, Kaohsiung, 80424 Taiwan; 2 Rapid Screening Research Center for Toxicology and Biomedicine, National Sun Yat-sen University, Kaohsiung, 80424 Taiwan; 3 Department of Obstetrics and Gynecology, Kaohsiung Medical University Hospital, Kaohsiung Medical University, Kaohsiung, 807 Taiwan; 4 Institute of Biomedical Sciences, National Sun Yat-sen University, Kaohsiung, 80424 Taiwan; 5 Graduate Institute of Medicine, College of Medicine, Kaohsiung Medical University, Kaohsiung, 807 Taiwan; 6 Department of Medical Research, E-Da Hospital/E-Da Cancer Hospital, I-Shou University, Kaohsiung, 82445 Taiwan

**Keywords:** Cancer, Structural biology

## Abstract

Mutant RAS genes play an important role in regulating tumors through lysine residue 104 to impair GEF-induced nucleotide exchange, but the regulatory role of KRAS K104 modification on the KRAS^G12D^ mutant remains unclear. Therefore, we simulated the acetylation site on the KRAS^G12D^ three-dimensional protein structure, including KRAS^G12D^, KRAS^G12D/K104A^ and KRAS^G12D/K104Q^, and determined their trajectories and binding free energy with GEF. KRAS^G12D/K104Q^ induced structural changes in the α2- and α3-helices, promoted KRAS instability and hampered GEF binding (ΔΔG = 6.14 kJ/mol). We found decreased binding to the Raf1 RBD by KRAS^G12D/K104Q^ and reduced cell growth, invasion and migration. Based on whole-genome cDNA microarray analysis, KRAS^G12D/K104Q^ decreased expression of NPIPA2, DUSP1 and IL6 in lung and ovarian cancer cells. This study reports computational and experimental analyses of Lys104 of KRAS^G12D^ and GEF, and the findings provide a target for exploration for future treatment.

## Introduction

KRAS is a small guanine nucleotide-binding protein (GTPase) that switches between an active form when GTP is bound and an inactive form when GDP is bound. The switch between the two forms is controlled by guanine nucleotide-exchange factors (GEFs) that induce activation by binding to Ras family proteins, including KRAS (and Rho-family GTPases), and catalyzing the release of GDP^1–3^. KRAS activity regulates tumor progression, differentiation, metastasis and survival^4^. Approximately 90% of KRAS missense mutations occur in codons 12 or 13^5^, and these mutations are associated with tumor formation^6,7^. KRAS is mutated in 25–30% of nonsmall cell lung cancer, of which c.35G>A (G12D) and codon 12 mutations are common in lung carcinoma (~ 18%)^8^ and ovarian cancer (~ 5.8%)^9^, respectively. The KRAS G12C mutation is not the most common, though it is frequently detected in colorectal and pancreatic cancers^10,11^. The c.35G>A mutation, from glycine to aspartic acid, interferes with GEF-induced GTP binding and activates KRAS downstream signaling^12^. Until recently, mutant KRAS was considered to be a therapeutic target in lung^13^ and ovarian cancer^14^.

In addition, acetylation negatively regulates KRAS signaling through GEF, as KRAS can be acetylated at lysine residue 104 (Lys104) to impair GEF-induced nucleotide exchange and inhibit oncogenic activity^15^. Yang et al. performed molecular dynamics (MD) simulations on a single KRAS structure. Their observations suggest that the K104Q mutation mimics K104 acetylation at the molecular level and completely disrupts the structural integrity of the α2-helix; therefore, the authors predicted that the KRAS–GEF interaction would be hindered by the mutation^15^. To better understand how the K104 modification alters the intrinsic biochemical properties of KRAS^G12D^, we focused on the KRAS/GEF protein complex structure and investigated its structural dynamics properties, including conformational changes, atomic fluctuations, and binding free energy differences between KRAS and GEF with KRAS^G12D/K104A^ and KRAS^G12D/K104Q^ mutants.

In this study, we predicted the three-dimensional (3D) structure of KRAS^G12D^, KRAS^G12D/K104A^ and KRAS^G12D/K104Q^ and analyzed the trajectory and binding free energy with GEF. We also characterized their cellular biological function and protein–protein interactions in cancer cell lines. This study reveals a novel pathway through which K104Q of KRAS^G12D^ may be useful for human cancer therapy.

## Materials and methods

### Structural predictions of KRAS and the KRAS/GEF complex

The 3D structures of KRAS and the KRAS/GEF complex were predicted using the MODELLER program (version 9v8)^16^. Human KRAS complexed with a GTP analog (PDB ID: 3GFT, KRAS-Q61H mutant structure) served as the template for modeling the wild-type (WT), G12D, G12D/K104A and G12D/K104Q KRAS structures. The crystal structure of the ternary Ras:SOS complex (PDB ID: 1XD2)^17^ served as the template for modeling the KRAS structures complexed with GEF. A Ramachandran plot for each structural model was prepared to assess quality^18^. The steepest descent algorithm^19^ was used for structural optimization, and PyMOL^20^ was used to display the 3D structures and charge distributions.

### Molecular dynamics simulations

Molecular dynamics (MD) simulations were performed using GROMACS version 5.1.4 software^21^. The OPLS-AA force field was used for energy calculations. Water was described using the TIP3P model, and the models were simulated in a cubic box with periodic boundary conditions. The energy of each model was minimized using the steepest descent algorithm. Each simulation was performed in canonical ensembles (NVT) followed by isothermal isobaric ensembles (NPT) with a position-restrained MD simulation for 100 ps. The MD simulations were performed at constant pressure and temperature for 50 ns using an integration timestep of 2 fs. The cutoff for nonbonded interactions was 10 Å. The coordinates from the MD simulations were saved every 100 timesteps.

### Trajectory analyses

To understand how amino acid replacements impact the structural and functional characteristics of Lys104 in KRAS, the trajectories of WT and mutant KRAS/GEF complex structures were analyzed for the following structural properties as a function of time. (1) The root mean square deviation (RMSD) of each KRAS backbone atom with respect to its position in the starting conformation, *r*^*ref*^, was calculated as $$RMSD(t) = \left[ {\frac{1}{M}\sum\limits_{{i = 1}}^{N} {m_{i} } \left| {r_{i} (t) - r_{i}^{{ref}} } \right|^{2} } \right]^{{1/2}}$$ where $$M={\sum }_{i}{m}_{i}$$ and $${r}_{i}(t)$$ reflect the position of atom *i* at time *t* after the last square fitting of the structure to the reference structure. (2) The conformational change of the α2 helix (which is located in the GEF-binding region) and the α3 helix of the WT and mutant structures derived from the equilibrated conventional MD trajectories as a function of the Cα dihedral angles of Met67 and Thr74 (both of which are located in the α2 helix) and Lys104 and Thr87 (both of which are located in the α3 helix). (3) Debye–Waller factors (B-factors) for Cα atoms, which reflect the fluctuation of the atoms about their average positions; the average positions for the Cα atoms were calculated using the coordinates of the last 2 ns of the MD trajectories. B-factors were calculated using the average RMSF values according to the relationship $$B-factor=\frac{8}{3}\times {\pi }^{2}\times {RMSF}^{2}$$. The RMSF is a measure of the deviation between the position of particle *i* and a reference position as $${RMSF_{i}} = \left[ {\frac{1}{T}\sum _{{t_{j} = 1}^{T} }\left| {{r_{i}} \left( {t_{j} } \right) - {r_{i}}^{{ref}} } \right|^{2} } \right]^{{{\raise0.7ex\hbox{$1$} \!\mathord{\left/ {\vphantom {1 2}}\right.\kern-\nulldelimiterspace} \!\lower0.7ex\hbox{$2$}}}}$$ where *T* is the time over which the RMSF is averaged and $${r}_{i}^{ref}$$ is the reference position of particle *i*. A large B-factor indicates a highly flexible atom.

### Calculation of the free energy of binding

The hybrid topologies of the systems for the free energy calculations were constructed using the pmx tool^22,23^. This tool automatically generates hybrid structures and topologies for amino acid mutations that represent the two physical states of the system. The double system (combining both branches of the thermodynamic cycle) in a single simulation box was set to keep the system neutral at all times during a transition^22^. After obtaining this hybrid structure, we used GROMACS to perform free energy simulations. One hundred snapshots were extracted from the 10-ns equilibrated trajectories, and a rapid 100-ps simulation was performed starting from each frame. Lambda (λ) ranged from 0 to 1 and from 1 to 0 for the forward and backward integrations, respectively, thus describing the interconversion of the WT to the mutant system and of the mutant to the WT system, respectively. Finally, the pmx tool was used to subsequently estimate free energy differences and integrate the multiple curves by the fast-growth thermodynamic integration approach, which relies on the Crooks–Gaussian Intersection (CGI) protocol^24^. In such a double-system/single-box setup, the estimated free energy corresponds to the ΔΔ*G* of binding.

### Western blotting and immunoprecipitation

Protein was extracted from cells in lysis buffer that contained a protease inhibitor cocktail (Roche Applied Science). Protein concentrations were determined using reagents from a BCA Protein Assay kit (Pierce, Bonn, Germany). For immunoprecipitation experiments, 2 mg of cell lysate was incubated with specific antibodies and immobilized on protein A/G PLUS-agarose beads (Santa Cruz Biotechnology). Proteins were separated by SDS-PAGE, transferred to a nitrocellulose membrane and blotted with specific antibodies, including those against RAS (1:300, Thermo Fisher Scientific, #16117)^25^, GEF H1 (1:500, Abcam, ab155785)^26^, RASA1 (1:500, Abcam, ab2922)^27^, Ac-lysine (1:500, Abcam, ab21623)^28,29^, phospho-AKT (Ser-473, 1:500, Cell Signaling, #4051)^30^, AKT (1:500, Cell Signaling, #2920)^31^ and β-actin (1;1000, Thermo Fisher Scientific, #AM4302)^32^, for western blotting.

### Pull-down assay and detection of active Ras

To evaluate the amount of active guanosine triphosphate − bound Ras, we used an Active Ras Pull-Down and Detection kit (Thermo Fisher Scientific). In brief, cells were homogenized in lysis buffer and incubated with GST-Raf1-RBD agarose beads at 4 °C for 1 h. The beads were washed with PBS, and the eluate was immunoblotted using K-, N- and H-Ras-specific antibodies.

### Cell number determination

Cell numbers in culture were analyzed using a Cell Counting Kit (CCK)-8 kit (Dojindo Laboratory, Japan). Briefly, 1 × 10^4^ cells were seeded into the wells of 96-well plates, and at the indicated times, the culture medium was replaced with CCK-8 reagent. The absorbance of each sample was measured at 450 nm and used to determine the number of viable cells in each culture. Values are presented as the mean ± standard deviation from three independent experiments.

### Wound-healing and invasion assays

For wound-healing assay, 5 × 10^5^ cells were seeded in the wells of 6-well plates and cultured for 24 h. Each cell layer was scratched with a new, sterile 200-μl pipette tip. After an additional 24 h, the extent of wound healing was assessed by visualization under a microscope. Invasion assays were performed in a 24-well Transwell invasion chamber (Corning, 8 μm) that was coated with Matrigel (BD Bioscience) on the upper chamber. The top chamber of each well was seeded with 5 × 10^4^ cells. After 24 h, the lower membrane surface was fixed with methanol and stained with 0.1% crystal violet. Invading cells were observed by microscopy.

### Human oligonucleotide DNA microarray

Human Whole Genome OneArray v7 (Phalanx Biotech Group, Taiwan) containing 29,204 DNA oligonucleotide sense probes of 50–60 nucleotides in length was recommended based on previous studies and used according to the manufacturer’s instructions. A total of 28,264 probes and 940 controls corresponding to the annotated genes from the RefSeq v70 and Ensembl v80 databases were included.

### Reverse transcription (RT)-PCR

RNA was isolated from cell lines using TRIZOL reagent (Invitrogen). cDNA was synthesized using a cDNA Synthesis kit (Promega, Madison, WI, USA). Quantitative PCR was performed with 10 ng of cDNA using Power SYBR Green PCR Master Mix (Applied Biosystems), and the cRNA was analyzed with an RT-PCR System kit (StepOne, Applied Biosystems, Darmstadt, Germany); 18S was used as the reference gene for normalization. All values were calculated using the 2^−ΔΔCT^ method.

### Transfection assay

Cells (1 × 10^5^) were seeded in a 6-well plate and incubated at 37 °C for 24 h. Plasmids were transfected with TurboFect transfection reagent (Fermentas, Hanover, MD) following the manufacturer’s protocol. pBabe-KrasG12D was purchased from Addgene (#58902).

## Results

### Modeling of the KRAS and KRAS/GEF complex

**Figure 1:**
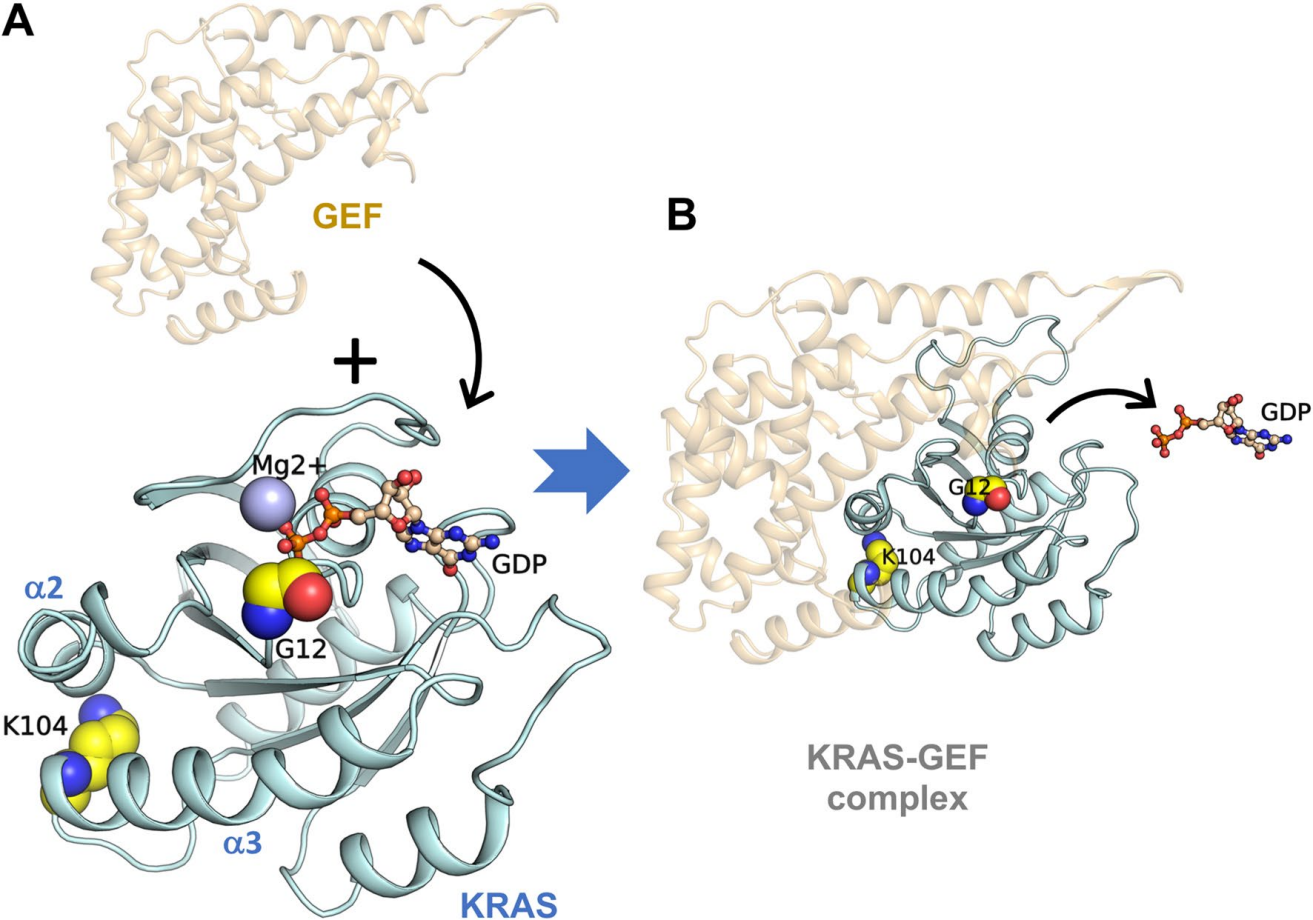
Modeled structures of KRAS and the KRAS/GEF complex. (**A**,**B**) Both KRAS (**A**) and the KRAS/GEF complex (**B**) are shown. The side chains of the residues to be mutated, Gly12 and Lys104, are represented by yellow spheres. GDP and Mg^2+^ are shown as a ball-and-stick model and a sphere, respectively. The figures were generated by PyMOL^20^.

The KRAS model was constructed using the published human KRAS crystal structure (PDB ID: 3GFT) as the template (Fig. 1A). Of the two replaced residues, Gly12 is located at the GDP-binding site, and Lys104 is located at the C-terminal end of the α3 helix, which is near the GEF-binding domain. Figure 1B shows the KRAS/GEF complex that was constructed by using the crystal structure of a ternary Ras:SOS (PDB ID: 1XD2) from *Homo sapiens* as the template. GEF activates monomeric KRAS by stimulating the release of GDP to allow binding of GTP.

### Structural changes in the WT and mutant KRAS/GEF complex in conventional MD simulations

**Figure 2:**
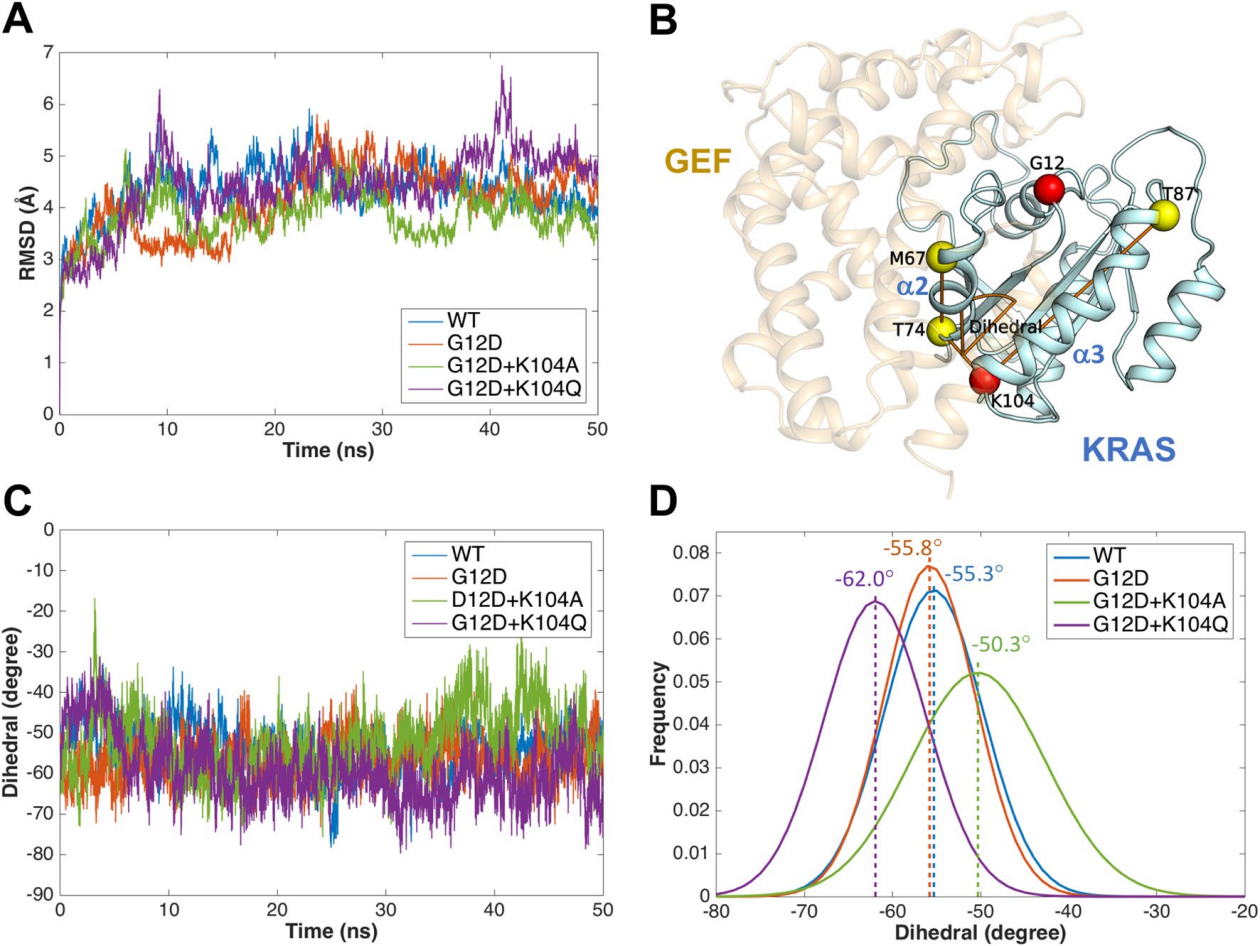
Analysis of the structural change of the WT and mutant KRAS/GEF complex in conventional MD simulations. (**A**) RMSD plot of the WT and mutant KRAS/GEF complex. (**B**) Schematic illustrating calculation of the dihedral angle. The conformational change of the α2- and α3-helices was detected by measuring the dihedral angle of Cα in Met67, Thr74, Lys104 and Thr87. (**C**) The dihedral angle of the α2- and α3-helices as a function of time. (**D**) The dihedral angle distributions in the last 30 ns of the simulation for the α2- and α3-helices are colored blue, red, green and purple for the WT, G12D, G12D + K104A and G12D + K104Q KRAS/GEF complexes, respectively. Dashed lines are mean values. The figures were generated by MATLAB R2015b and PyMOL^20^.

The trajectories from MD simulations of the WT and mutant KRAS/GEF complex in explicit solvent models were calculated. The RMSD values of the backbone atoms for the WT and mutant KRAS/GEF complex with regard to the initial structures were plotted (Fig. 2A), and the quality and convergence of the simulated dynamic trajectories can be estimated by assessing the RMSD values. With regard to the simulations of the WT and mutant KRAS/GEF complex, the data obtained demonstrated that after a rapid increase during the first 0.2 ns, the trajectories were stable, with average values of 4.36, 4.14, 3.84 and 4.44 Å for the WT, G12D, G12D + K104A and G12D + K104Q KRAS/GEF complexes, respectively. In contrast, the RMSD values showed a large increase as a function of time for the G12D + K104Q KRAS/GEF complex (Fig. 2A). The results reveal that the conformational changes of the G12D + K104Q KRAS/GEF complex were significant when compared with other complexes.

To understand the structural changes of KRAS in the GEF-binding region caused by these mutations, we detected the conformational difference between the WT and mutant KRAS/GEF complex structures by measuring the dihedral angle of the α2-helix and α3-helix (Cα in Met67, Thr74, Lys104 and Thr87) (Fig. 2B). Figure 2C shows the dihedral angle of the α2-helix and α3-helix as a function of time. In Fig. 2D, histograms of the dihedral angle during the last 30 ns of the simulations are depicted; the average dihedral angles are − 55.3°, − 55.8°, − 50.3° and − 62.0°. As illustrated in Fig. 2D, the most populated dihedral angle of the α2-helix and α3-helix in the WT (blue) and G12D (red) KRAS/GEF complexes shows a similar distribution of the degree. The most populated dihedral angle of the G12D + K104A KRAS/GEF complex (green) is ~ 5° larger and more widely distributed than that in WT KRAS (blue). That is, the mutation causes the region of α2 and α3 helices of the G12D + K104Q KRAS/GEF complex to be more flexible. Interestingly, a different phenomenon was observed for the G12D + K104Q KRAS/GEF complex (purple), where a left-shifted distribution of the dihedral angle of the α2- and α3-helices was detected by comparison with the WT KRAS/GEF complex (blue), with the middle value being ~ 7° smaller in the G12D + K104Q KRAS/GEF complex (purple) (Fig. 2D). The results suggest that more structural changes of the α2- and α3-helices were prevalent in the G12D + K104Q KRAS/GEF complex, which is consistent with the RMSD results (Fig. 2A). The K104Q mutation induced an additional structural change in the α2- and α3-helices, which may be involved in the support and stabilization of GEF binding. Based on these analyses, the KRAS K104Q mutation is predicted to affect GEF binding.

### Residue fluctuation of the WT and mutant KRAS/GEF complex structures

**Figure 3:**
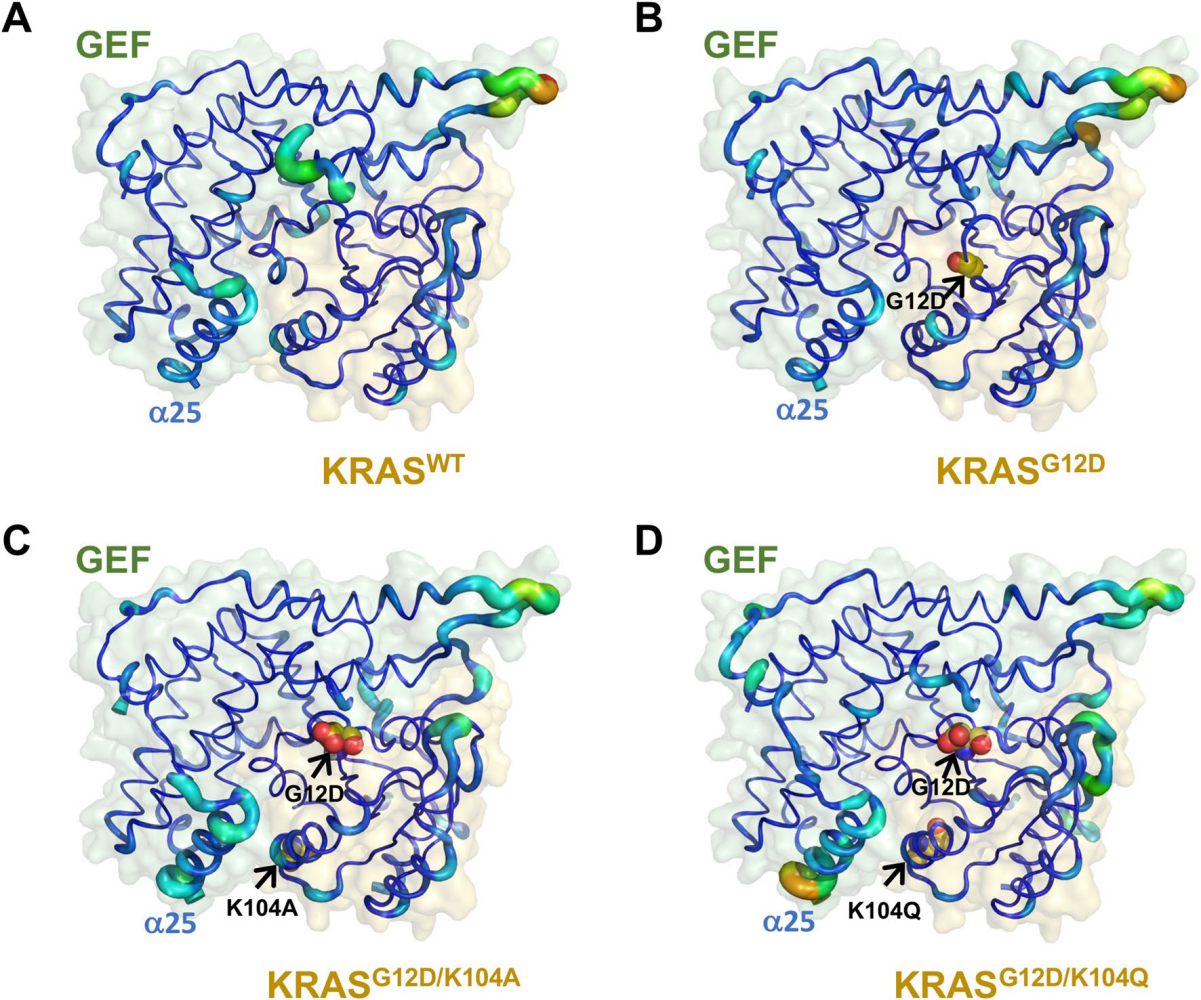
Analysis of atomic fluctuations. (**A**–**D**) KRAS/GEF complex structures of (**A**) WT and (**B**) G12D, (**C**) G12D/K104A and (**D**) G12D/K104Q mutant KRAS are shown as cartoon putty representations; blue represents the lowest and red the highest B-factor value. In addition, the size of the tube reflects the value of the B-factor, whereby the larger is the B-factor, the thicker is the tube. The figures were generated by PyMOL^20^.

The results of B-factor calculations for each residue revealed that the atomic fluctuations of the G12D/K104Q mutant were significant at the KRAS/GEF interaction regions (especially in the α25-helix of the GEF protein) when compared with WT and with the G12D and G12D/K104A mutants (Fig. 3). This is consistent with the left-shift distributed dihedral angle of the G12D/K104Q mutant relative to WT and the other two mutants. The data from MD simulations of the KRAS/GEF complex suggest that the K104Q mutation leads to a considerable decrease in the affinity of KRAS for GEF.

### Analysis of KRAS/GEF interactions

**Figure 4:**
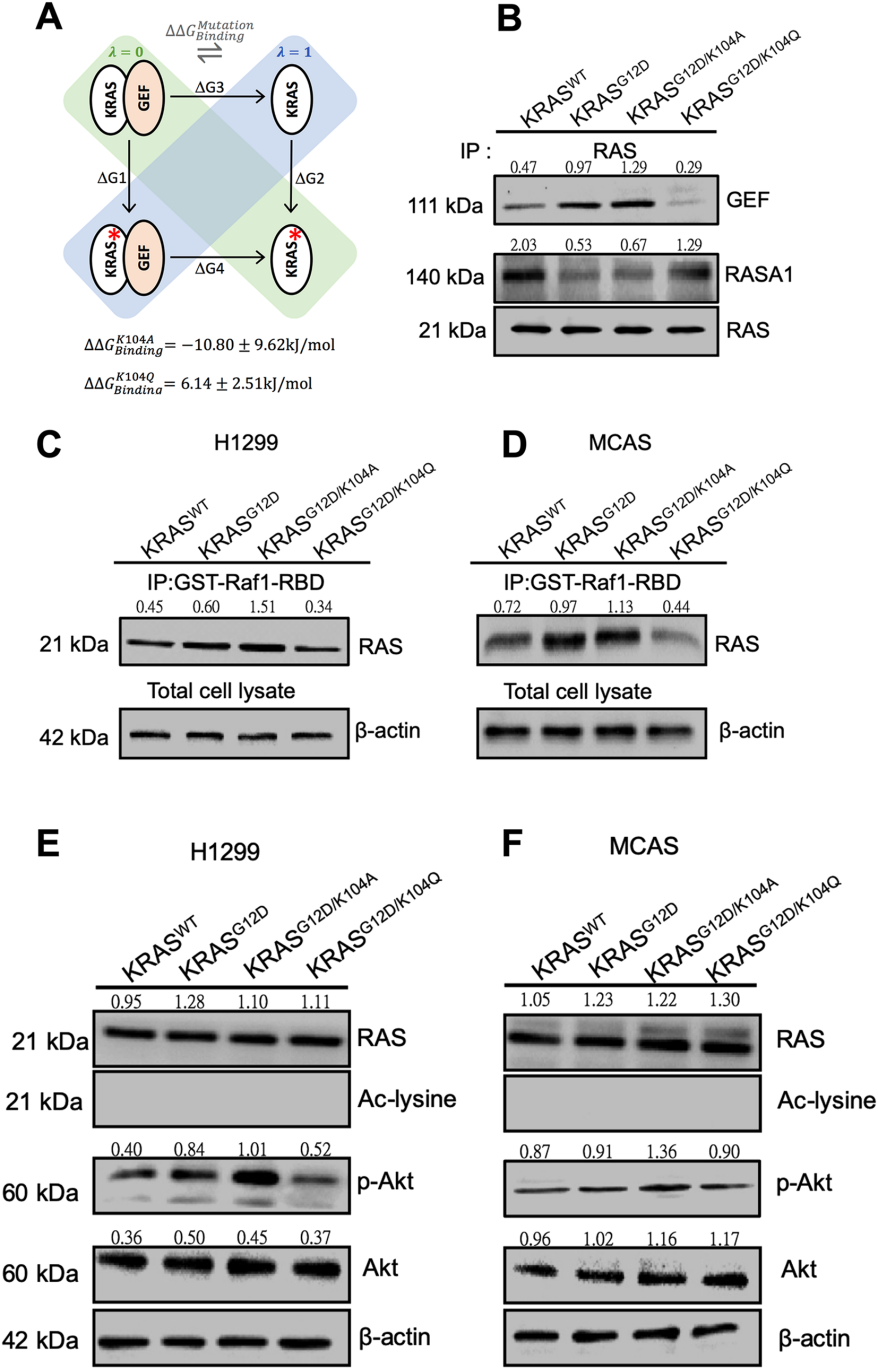
Analysis of KRAS/GEF interactions. (**A**) Two branches of a thermodynamic cycle are placed in one simulation box. The different boxes in the scheme are indicated by the green ($\lambda =0$) and blue ($\lambda =1$) shading. The free energy estimate corresponds to a double free energy difference: ΔΔ*G* = Δ*G1* – Δ*G2*. The figure was generated by Microsoft PowerPoint 2016. (**B**) Interactions of KRAS with GEF and RASA1. KRAS was detected by western blot analysis after immunoprecipitation of endogenous GEF and RASA1 in H1299 cells after transfection with KRS^WT^, KRAS^G12D^, KRAS^G12D/K104A^ or KRAS^G12D/K104Q^ plasmids. (**C**–**F**) Relative levels of GST-Raf1-RBD, RAS, Ac-lysine, p-AKT and AKT detected by western blotting after overexpression of KRAS^G12D^, KRAS^G12D/K104A^ or KRAS^G12D/K104Q^ plasmids in (**C**,**E**) H1299 (KRAS^WT^) and (**D**,**F**) MCAS (KRAS^MT^) cancer cell lines. β-Actin was used as a loading control for cell lysates. The full-length and multiple exposures blots are presented in Supplementary Information.

KRAS/GEF binding free energy differences between the G12D, G12D + K104A and G12D + K104Q mutants were calculated in a series of alchemical free energy simulations using the CGI protocol^24^. The GEF binding affinity differences (${\Delta \Delta G}_{Binding}^{Mutation}$) were calculated according to Δ*G*_1_ (GEF-bound) – Δ*G*_2_ (GEF-free), as shown in the thermodynamic cycle that was distributed between two simulation boxes for λ = 0 (green) and λ = 1 (blue; Fig. 4A). Thus, the change in the net charge during the alchemical simulation remains zero. Notably, stabilizing mutations have negative ΔΔ*G* values. We adopted the CGI protocol to calculate the binding affinity differences between KRAS and GEF and the single point mutations. The resulting binding free energy differences (${\Delta \Delta G}_{Binding}^{Mutation}$) between G12D KRAS and G12D + K104A KRAS and between G12D KRAS and G12D + K104Q KRAS were − 10.80 and 6.14 kJ/mol, respectively (Fig. 4A). The results reveal that the KRAS-GEF interaction may be disrupted in the G12D + K104Q mutant (i.e., ${\Delta \Delta G}_{Binding}^{Mutation}>0$), and thus, GEF may not be able to activate this KRAS mutant by stimulating the release of GDP to allow binding of GTP.

In addition to the alchemical free energy calculation, we examined the level of protein–protein interaction between GEFs, effectors and KRAS by experimental methods. We used immunoprecipitation—western blotting and pull-down assays and detection of active Ras when KRAS^WT^, KRAS^G12D^, KRAS^G12^^D/K104A^ and KRAS^G12D/K104Q^ were individually expressed in cell lines. KRAS^G12D/K104A^ and KRAS^G12D/K104Q^ plasmids were generated with pBabe-Kras^G12D^ (Addgene) using site-directed mutagenesis. Active Ras binds to the Ras-binding domain (RBD) of Raf1, leading to Ras activation. The results of the analysis showed increased interaction of KRAS^G12D/K104Q^ with Ras GTPase-activating protein 1 (RASA1, RasGAP) but decreased interaction with GEFs (Fig. 4B) as well as effectors, reducing the binding to RBD of Raf1 and leading to Ras inactivation (Fig. 4C,D) compared with KRAS^G12D^ and KRAS^G12D/K104A^. We also analyzed the level of RAS in the total MCAS and H1299 cell lysates, RAS downstream pathways (phospho-AKT) and acetylation. According to the results, the levels of RAS of KRAS^WT^, KRAS^G12D^, KRAS^G12D/K104A^ and KRAS^G12D/K104Q^ were the same. However, ac-lysine of RAS was not detected. KRAS^G12D^ and KRAS^G12D/K104A^ exhibited increased levels of p-AKT compared to WT and KRAS^G12D/K104Q^ (Fig. 4E,F).

### ***K104 modification affects KRAS***^***G12D***^***-mediated cell growth and motility***

**Figure 5:**
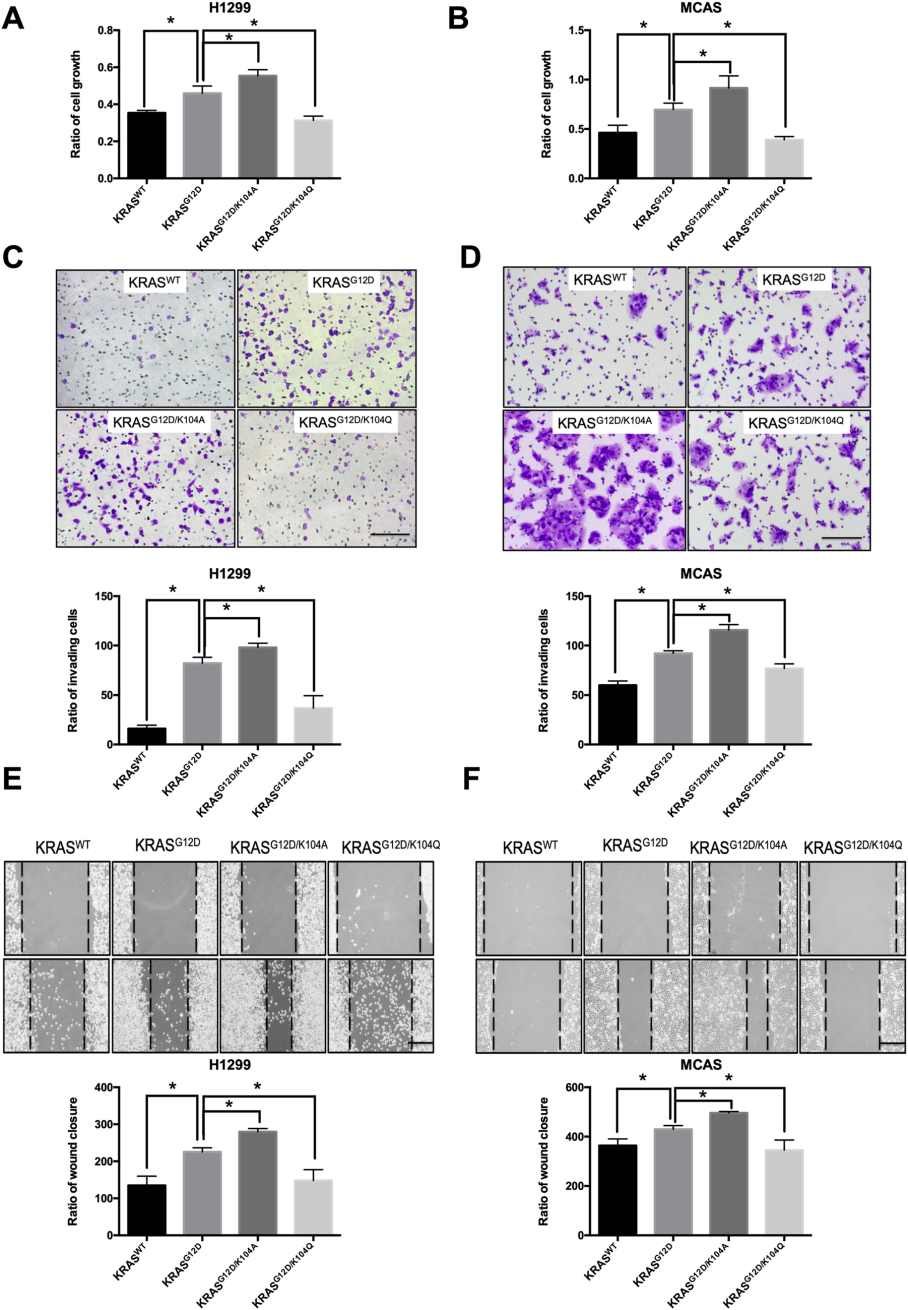
Cellular function of K104 KRAS^G12D^ in lung and ovarian cancer. The (**A**,**C**,**E**) H1299 lung cancer cell line and (**B**,**D**,**F**) MCAS ovarian cancer cell line were transfected with KRAS^WT^ (1 μg), KRAS^G12D^ (1 μg), KRAS^G12D/K104A^ (1 μg) or KRAS^G12D/K104Q^ (1 μg). After 24 h, (**A**,**B**) cell growth was analyzed by CCK-8 assays, (**C**,**D**) invasion ability was analyzed with an invasion chamber, and (**E**,**F**) migration ability was analyzed by a wound-healing assays (**E**,**F**). Data are the mean ± SD from three independent experiments. *P < 0.05 *vs*. untreated control; two-tailed Student’s *t-*test. Scale bar = 200 μm.

To investigate whether K104 modification affects KRAS^G12D^, we used KRAS^WT^, KRAS^G12D^, KRAS^G12D/K104A^ and KRAS^G12D/K104Q^ and analyzed the biological function of these mutations in lung and ovarian cell lines. Individual expression of KRAS^G12D^, KRAS^G12D/K104A^ and KRAS^G12D/K104Q^ was induced by transfection of plasmid DNA in H1299 and MCAS cancer cell lines. We then analyzed their effects on cell growth (Fig. 5A,B), wound healing (Fig. 5C,D) and invasion (Fig. 5E,F). KRAS^G12D^ and KRAS^G12D/K104A^ promoted cell growth, wound healing and invasion, whereas KRAS^G12D/K104Q^ attenuated these processes. Moreover, compared to KRAS^G12D^, KRAS^G12D/K104A^ significantly increased the rate of cell growth (P = 0.019) and migration ability in H1299 (P < 0.0001) and MCAS (P = 0.02) cell lines. These results suggest that K104 modification regulates KRAS^G12D^-mediated cell growth and motility in lung and ovarian cancer cell lines.

### ***KRAS***^***G12D/K104Q***^*** mediates NPIPA2, DUSP1 and IL6 expression***

**Figure 6:**
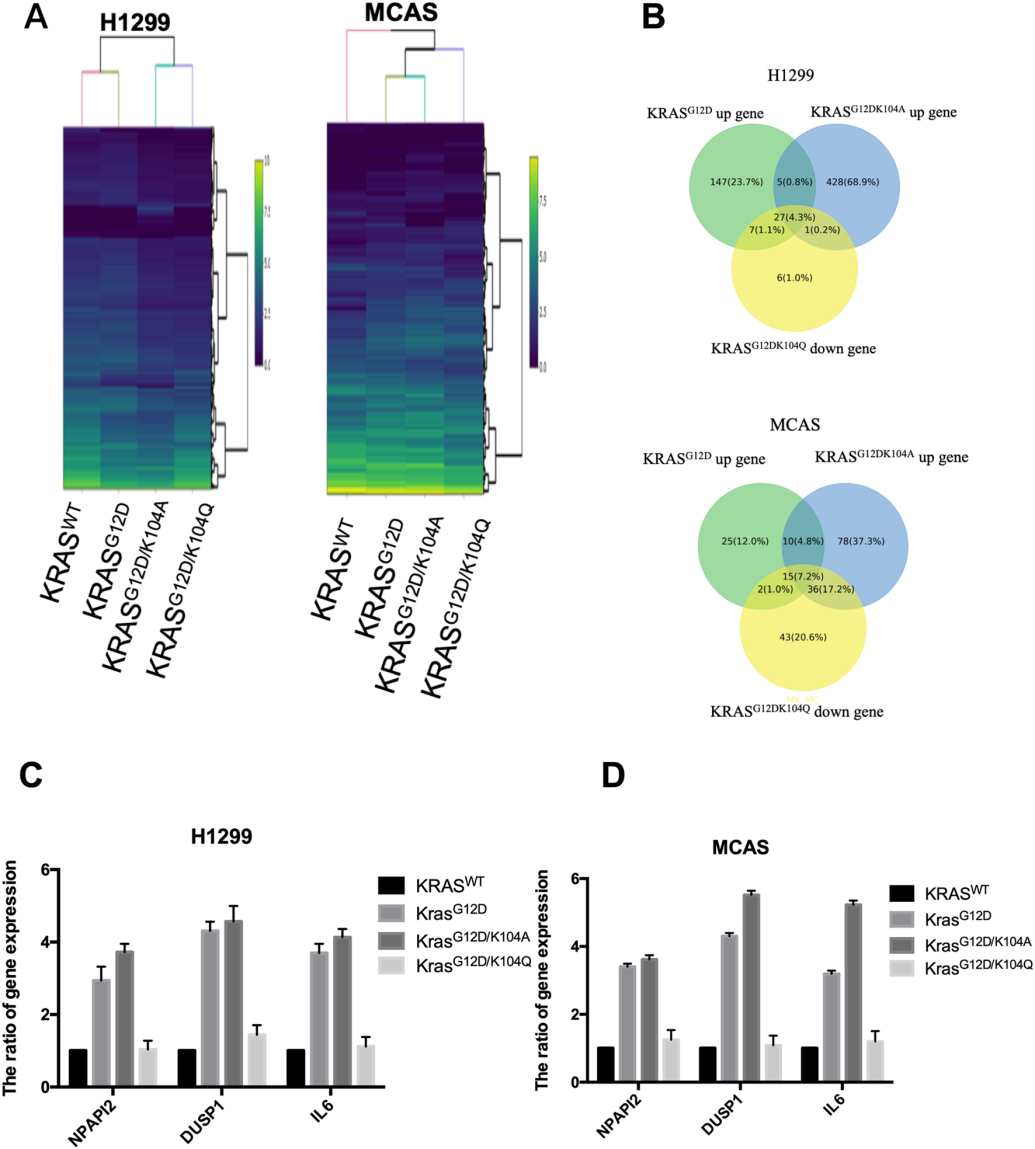
KRAS^G12DK104Q^ inhibits NPIPA2, DUSP1 and IL6 expression compared to KRAS^G12D^ and KRAS^G12D/K104A^. (**A**) H1299 and MCAS cells were transfected with a KRAS^WT^ or KRAS^G12D^, KRAS^G12D/K104A^ or KRAS^G12D/K104Q^ plasmid. We analyzed global gene expression profiles using human oligonucleotide DNA microarrays with three independent RNA samples at 48 h posttransfection. The intensity of each spot was analyzed by GenePix 4.1 software (Molecular Devices). (**B**) Three identified genes, *NPIPA2, DUSP1* and *IL6*, were upregulated in the presence of KRAS^G12D^ and KRAS^G12D/K104A^ and downregulated in the presence of KRAS^G12D/K104Q^ compared with expression in the control group. (**C**,**D**) *NPIPA2, DUSP1* and *IL6* expression was detected by RT-PCR and after transfection of the mutant forms of KRAS. Data are the mean ± SD from three independent experiments. *P < 0.05 vs. untreated control; two-tailed Student’s *t*-test.

Our previous study found that KRAS^G12D/K104Q^ affects cellular biological functions by associating with GTP binding. However, the related downstream genes were not clear. Therefore, we sought to identify downstream genes of KRAS^G12D/K104Q^ that are reduced compared with KRAS^G12D/K104A^ using a whole-genome cDNA microarray to screen KRAS^G12D^ (> twofold), KRAS^G12D/K104A^ upregulation (> twofold) and KRAS^G12D/K104Q^ downregulation (> twofold) of gene expression in the lung cancer cell line H1299 and the ovarian cancer cell line MCAS (Fig. 6A). Further selection of the top three and four assessments of the microarray results showed that NPIPA2, DUSP1 and IL6 were consistently identified as upregulated genes in KRAS^G12D^-expressing cells and KRAS^G12D/K104A^-expressing cells and downregulated in KRAS^G12D/K104Q^-expressing cells relative to control cells (Fig. 6B). To confirm the microarray results, *NPIPA2, DUSP1* and *IL6* expression was examined by qRT-PCR, and expression was induced by KRAS^G12D^ and KRAS^G12D/K104A^ and decreased by KRAS^G12D/K104Q^ in cancer cell lines (Fig. 6C,D). These results were obtained in both cell lines, with the three genes showing the same pattern in both. These results suggest that KRAS^G12D/K104Q^ decreases expression of NPIPA2, DUSP1 and IL6 in lung and ovarian cancer cells.

## Discussion

Based on the crystal structure of KRAS bound to GDP (PDB code 4LPK)^33^, K104 is located on the C-terminal end of the α3-helix, and the positively charged side chain directly interacts with a carbonyl group at the negative pole of the α2-helix. These interactions have been predicted to play a key role in the structural stability of the KRAS-GEF complex^15^. Previous studies have indicated that K104 modification may affect both guanine nucleotide exchange and GTP hydrolysis rates and influence the downstream signal transduction pathways involved in the control of cell survival. Marcus et al*.* showed that HRAS K104Q decreases intrinsic and catalyzed hydrolytic behavior but that K104A does not affect intrinsic hydrolysis^34^. Based on the NIH3T3 proliferation assay, Yang et al*.* observed that the G12V and G12V/K104A forms of KRAS enhanced cell survival but that G12V/K104Q did not^15^. Knyphausen et al*.* also observed that KRAS G12V/K104Q displayed a significantly reduced SOS-catalyzed nucleotide exchange rate compared to KRAS G12V^28^. Yin et al*.* reported that KRAS K104Q exhibits defects in both GEF-mediated exchange and GAP-mediated GTP hydrolysis and also indicated that KRAS K104Q did not alter steady-state GTP-bound levels or the ability of the oncogenic KRAS G12V mutant in NIH3T3 mouse fibroblasts^35^. From these findings, it is clear that these investigations focused on the K104 modification of RAS-WT or RAS-G12V; however, very few attempts have been made to target RAS-G12D. In this study, we focused on the KRAS-G12D mutant and investigated how KRAS K104 modification affects the KRAS^G12D^-GEF complex interaction and mediates cell growth and motility. Based on our results, K104Q is able to reduce GTP-bound levels of KRAS-G12D and the oncogenic effects of the KRAS-G12D mutant in H1299 and MCAS cells, but K104A cannot.

MD simulations of the KRAS/GEF protein complex structure revealed that the conformational changes of the G12D + K104Q mutant were significant at the α2- and α3-helices when compared with the WT, G12D and G12D + K104A KRAS/GEF complexes (Fig. 2). Moreover, the K104Q mutation may induce additional fluctuations at the KRAS/GEF interaction regions (especially in the α25-helix of the GEF protein) (Fig. 3). As mentioned above, the α2- and α3-helices play important roles in the binding of GEFs; therefore, we postulate that such fluctuations may promote instability in this region, which consequently reduces the binding ability between KRAS and GEFs. The data obtained from the binding free energy differences indicate that the binding of GEF with the K104Q mutant is less favorable than that of GEF with the K104A mutant (Fig. 4A). These results are consistent with previous studies^15,28,35^ showing that KRAS K140Q decreases the SOS-catalyzed nucleotide exchange rate.

In this study, we found that the effect of the mutant G12D/K104A is higher than that of G12D. This phenomenon was confirmed in both our computational and experimental analyses. Our computational studies showed that the G12D and G12D/K104A forms of the KRAS-GEF complex have different effects on the interactive regions (α2- and α3-helices). According to RMSD results, the most populated dihedral angle of the G12D + K104A KRAS/GEF complex (green) is ~ 5° larger and more widely distributed than that in G12D KRAS (red) (Fig. 2D). Additionally, the data obtained from the binding free energy differences indicate that the binding of GEF with the G12D/K104A mutant is more favorable than that of GEF with the G12D mutant (ΔΔG = − 10.80 kJ/mol) (Fig. 4A). This result is also consistent with our immunoprecipitation assay (Fig. 4B). Moreover, our experimental studies showed that G12D/K104A slightly increased the active form of KRAS compared with the G12D mutant (Fig. 4C,D). Therefore, we hypothesize that the G12D/K104A mutant allows GEF to have a greater ability to activate KRAS by removing GDP than the G12D mutant. As a result, the G12D/K104A mutant may have a more aggressive oncogenic phenotype than the G12D mutant. Regardless, the detailed mechanisms need to be further investigated.

We also show that KRAS^G12D/K104Q^ induces structural changes in the α2-/α3-helix to block binding with GEF and mediate tumor formation. KRAS^G12D/K104Q^ also induced expression of the downstream target genes NPIPA2, DUSP1 and IL6. A previous study suggested that Lys104 of KRAS is easily acetylated by posttranslational modification^15^ to regulate cellular biological function. KRAS^G12V^ K104 acetylation (as represented by K104Q) represses downstream signaling and inhibits cell growth through HDAC6 and SIRT2 in NIH 3T3 cells^36^. Previous studies as well as our own observed that KRAS^G12V^ and KRAS^G12D^ have the same function with respect to regulating the KRAS pathway and cellular function when K104 is acetylated. Although KRAS^G12D^ can activate cancer cells, progression to malignancy requires additional genetic lesions^37^. Among cancers with the highest mortality rates, KRAS-activating mutations occur in ~ 90% of pancreatic cancers, 40% of colon cancers and 30% of lung cancers. Therefore, it is important to consider cancer therapies that target KRAS mutations. Research has predominantly focused on EGFR mutation for lung cancer-related drug development, and there are many EGFR antagonists that have been used for clinical treatment. Unfortunately, the mortality rate for lung cancer remains high throughout the world. Indeed, epidemiological statistics indicate that EGFR and KRAS mutations are present in ~ 50% of lung adenocarcinomas, of which ~ 20% are EGFR mutations and 26% KRAS mutations^38^. KRAS mutations occur in 3.7–36.4% of endometrioid ovarian cancers^39,40^, though therapies targeting KRAS have rarely been used to treat ovarian cancer. Hence, it is very important to focus on KRAS mutations for the development of future therapies to treat lung and ovarian cancer.

Although expression of NPIPA2, DUSP1 and IL6 was decreased after acetylation of KRAS, it is still unclear whether this is related to the ubiquitin system. This possibility will be pursued in our future studies. Finally, our findings suggest that KRAS^G12D/K104A^ regulates GEF to activate KRAS signaling and promote cell growth, invasion and migration in lung and ovarian cancer cell lines. Mutationally activated KRAS (through G12D and G12V) is still a difficult pharmacological target. Next, we will analyze K104 acetylation of KRAS^G12D^ using histone deacetylase inhibitors (HDACis), which regulate cellular functions, including cell growth, invasion, migration and apoptosis, by repressing expression of HDAC, leading to increased acetylation of lysine residues in target proteins^41,42^. The HDACi trichostatin A (TSA) is a naturally derived hydroxamic acid, which has been studied as a potential nontoxic anticancer drug^43,44^. It is hoped that an HDACi such as TSA—either alone or in combination with other chemotherapy approaches—may provide a viable strategy for clinical targeting options in lung and ovarian cancer.

## Supplementary information

Supplementary file1.

## Data Availability

The raw microarray data have been submitted to GEO database under accession number GSE158235 (https://www.ncbi.nlm.nih.gov/geo/query/acc.cgi?acc=GSE158235).

## References

[1] Vigil D, Cherfils J, Rossman KL, Der CJ (2010). Ras superfamily GEFs and GAPs: Validated and tractable targets for cancer therapy?. Nat. Rev. Cancer.

[2] Bos JL, Rehmann H, Wittinghofer A (2007). GEFs and GAPs: Critical elements in the control of small G proteins. Cell.

[3] Schmidt A, Hall A (2002). Guanine nucleotide exchange factors for Rho GTPases: Turning on the switch. Genes Dev..

[4] Karnoub AE, Weinberg RA (2008). Ras oncogenes: Split personalities. Nat. Rev. Mol. Cell Biol..

[5] Janakiraman M (2010). Genomic and biological characterization of exon 4 KRAS mutations in human cancer. Can. Res..

[6] Engelman JA (2008). Effective use of PI3K and MEK inhibitors to treat mutant Kras G12D and PIK3CA H1047R murine lung cancers. Nat. Med..

[7] De Roock W (2010). Association of KRAS p.G13D mutation with outcome in patients with chemotherapy-refractory metastatic colorectal cancer treated with cetuximab. JAMA.

[8] Wood K, Hensing T, Malik R, Salgia R (2016). Prognostic and predictive value in KRAS in non-small-cell lung cancer: A review. JAMA Oncol..

[9] Rahman MT (2013). KRAS and MAPK1 gene amplification in type II ovarian carcinomas. Int. J. Mol. Sci..

[10] Bailey P (2016). Genomic analyses identify molecular subtypes of pancreatic cancer. Nature.

[11] Giannakis M (2016). Genomic correlates of immune-cell infiltrates in colorectal carcinoma. Cell Rep..

[12] Chen CC (2013). Computational analysis of KRAS mutations: Implications for different effects on the KRAS p.G12D and p.G13D mutations. PLoS ONE.

[13] Naidoo J, Drilon A (2016). KRAS-mutant lung cancers in the era of targeted therapy. Adv. Exp. Med. Biol..

[14] Han C (2018). Novel targeted therapies in ovarian and uterine carcinosarcomas. Discov. Med..

[15] Yang MH (2012). Regulation of RAS oncogenicity by acetylation. Proc. Natl. Acad. Sci. U.S.A..

[16] Eswar, N. *et al.* Comparative protein structure modeling using Modeller. *Current protocols in bioinformatics/editoral board, Andreas D. Baxevanis ... [et al.]* **Chapter 5**, Unit 5 6, doi:10.1002/0471250953.bi0506s15 (2006). 10.1002/0471250953.bi0506s15 PMC4186674 18428767

[17] Sondermann H (2004). Structural analysis of autoinhibition in the Ras activator Son of sevenless. Cell.

[18] Laskowski RA, Macarthur MW, Moss DS, Thornton JM (1993). PROCHECK—A program to check the stereochemical quality of protein structures. J. Appl. Crystallogr..

[19] Arfken G (1985). The Method of Steepest Descents. 74 in Mathematical Methods for Physicists.

[20] PyMOL v1.5.0.5. Available: https://www.pymol.org.

[21] Van Der Spoel D (2005). GROMACS: Fast, flexible, and free. J. Comput. Chem..

[22] Gapsys V, Michielssens S, Seeliger D, de Groot BL (2015). pmx: Automated protein structure and topology generation for alchemical perturbations. J. Comput. Chem..

[23] Gapsys V, de Groot BL (2017). pmx webserver: A user friendly interface for alchemistry. J. Chem. Inf. Model.

[24] Goette M, Grubmuller H (2009). Accuracy and convergence of free energy differences calculated from nonequilibrium switching processes. J. Comput. Chem..

[25] Wu D (2018). Nogo-B receptor promotes epithelial-mesenchymal transition in non-small cell lung cancer cells through the Ras/ERK/Snail1 pathway. Cancer Lett..

[26] Zhao Y (2019). Microbial recognition by GEF-H1 controls IKKepsilon mediated activation of IRF5. Nat. Commun..

[27] Lei Z (2015). MicroRNA-132/212 family enhances arteriogenesis after hindlimb ischaemia through modulation of the Ras-MAPK pathway. J. Cell Mol. Med..

[28] Knyphausen P, Lang F, Baldus L, Extra A, Lammers M (2016). Insights into K-Ras 4B regulation by post-translational lysine acetylation. Biol. Chem..

[29] Yuan P (2020). Laminar flow inhibits the Hippo/YAP pathway via autophagy and SIRT1-mediated deacetylation against atherosclerosis. Cell Death Dis.

[30] Bell CM, Raffeiner P, Hart JR, Vogt PK (2019). PIK3CA cooperates with KRAS to promote MYC activity and tumorigenesis via the bromodomain protein BRD9. Cancers (Basel).

[31] Jones S (2020). Targeting of EGFR by a combination of antibodies mediates unconventional EGFR trafficking and degradation. Sci. Rep..

[32] Lee FHF, Zhang H, Jiang A, Zai CC, Liu F (2018). Specific alterations in astrocyte properties via the GluA2-GAPDH complex associated with multiple sclerosis. Sci. Rep..

[33] Ostrem JM, Peters U, Sos ML, Wells JA, Shokat KM (2013). K-Ras(G12C) inhibitors allosterically control GTP affinity and effector interactions. Nature.

[34] Marcus K, Johnson C, Sanchez J, Mattos C (2016). Crystal structures of acetylated HRas K104 mimic K104Q and mutant K104A suggest unique role of K104 in interlobe communication across HRas. Faseb J..

[35] Yin GW (2017). A KRAS GTPase K104Q mutant retains downstream signaling by offsetting defects in regulation. J. Biol. Chem..

[36] Yang MH (2013). HDAC6 and SIRT2 regulate the acetylation state and oncogenic activity of mutant K-RAS. Mol. Cancer Res..

[37] Tuveson DA (2004). Endogenous oncogenic K-ras(G12D) stimulates proliferation and widespread neoplastic and developmental defects. Cancer Cell.

[38] Dogan S (2012). Molecular epidemiology of EGFR and KRAS mutations in 3,026 lung adenocarcinomas: higher susceptibility of women to smoking-related KRAS-mutant cancers. Clin. Cancer Res..

[39] Cuatrecasas M (1998). K-ras mutations in nonmucinous ovarian epithelial tumors: A molecular analysis and clinicopathologic study of 144 patients. Cancer.

[40] Hogdall EV (2003). K-ras alterations in Danish ovarian tumour patients from the Danish “Malova” Ovarian Cancer study. Gynecol. Oncol..

[41] Finnin MS (1999). Structures of a histone deacetylase homologue bound to the TSA and SAHA inhibitors. Nature.

[42] Yoshida M, Kijima M, Akita M, Beppu T (1990). Potent and specific inhibition of mammalian histone deacetylase both in vivo and in vitro by trichostatin A. J. Biol. Chem..

[43] Mottamal M, Zheng S, Huang TL, Wang G (2015). Histone deacetylase inhibitors in clinical studies as templates for new anticancer agents. Molecules.

[44] Nervi C (2001). Inhibition of histone deacetylase activity by trichostatin A modulates gene expression during mouse embryogenesis without apparent toxicity. Can. Res..

