# Supplementary material for: KRAS K104 modification affects the KRASG12D-GEF interaction and mediates cell growth and motility
Source: Sci Rep. 2020 Oct 15;10:17447. doi: 10.1038/s41598-020-74463-5 (PMC7567070; doi:10.1038/s41598-020-74463-5)

KRAS K104 modification affects the KRAS^G12D^-GEF interaction and mediates cell growth and motility

A running title: KRAS K104 modification affects cell growth and motility

Chih-Chieh Chen^1,2^, Chia-Yi Hsu^3^, Hsiao-Yun Lin^3^, Hong-Qi Zeng^1^, Kuang-Hung Cheng^4^, Chia-Wei Wu^6^, Eing-Mei Tsai^3,5^* and Tsung-Hua Hsieh^6^*

^1^Institute of Medical Science and Technology, National Sun Yat-sen University, Kaohsiung, Taiwan

^2^Rapid Screening Research Center for Toxicology and Biomedicine, National Sun Yat-sen University, Kaohsiung, Taiwan

^3^Department of Obstetrics and Gynecology, Kaohsiung Medical University Hospital, Kaohsiung Medical University, Kaohsiung, Taiwan

^4^Institute of Biomedical Sciences, National Sun Yat-Sen University, Kaohsiung, Taiwan

^5^Graduate Institute of Medicine, College of Medicine, Kaohsiung Medical University, Kaohsiung, Taiwan

^6^Department of Medical Research, E-Da Hospital/ E-Da Cancer Hospital, I-Shou University, Kaohsiung, Taiwan.

*Correspondence: Tsung-Hua Hsieh, PhD, Tel.: 886-7-6151100 ext 5072, Fax: 886-7-311-2493, E-mail address: pelagice@yahoo.com.tw, Mailing address: 6 Yi-Da Rd., Yan-Chau District, Kaohsiung 82445, Taiwan

Eing-Mei Tsai, MD, PhD, Tel.: 886-7-3121101 ext 6424, Fax: 886-7-311-2493, E-mail address: [tsaieing@yahoo.com](mailto:tsaieing@yahoo.com), Mailing address: No. 100, Zihyou 1st Rd., Sanmin District, Kaohsiung City 807, Taiwan

Full-length gels and blots for figure 4B.


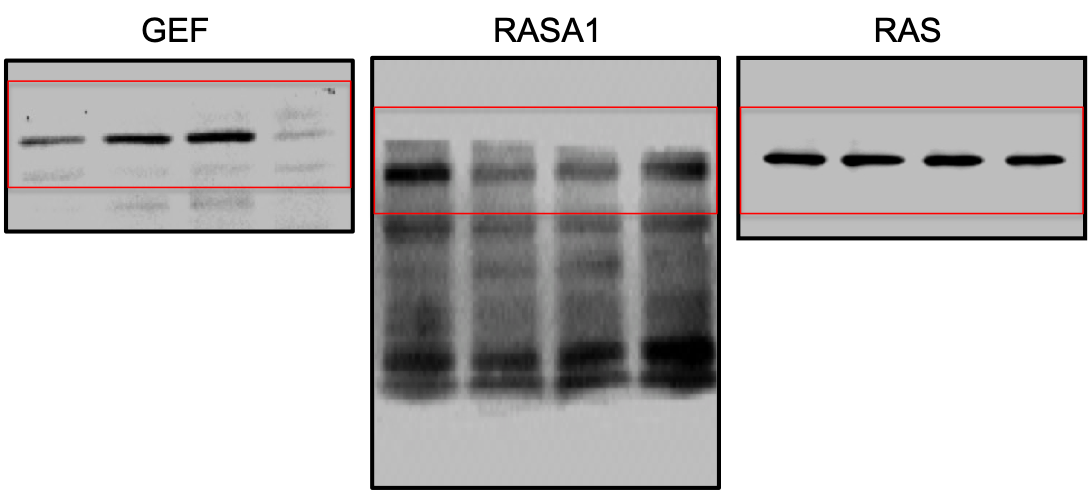


Multiple exposures


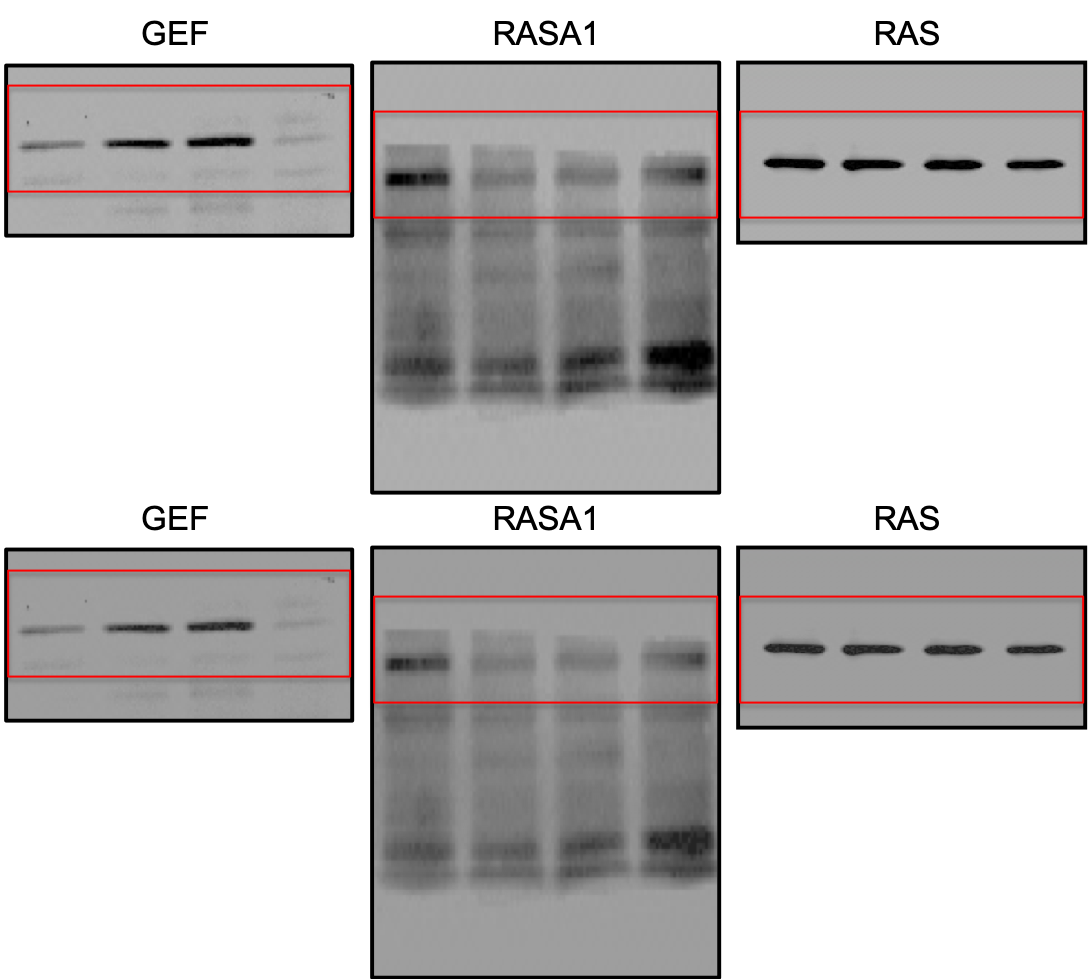


Full-length gels and blots for figure 4C and D.


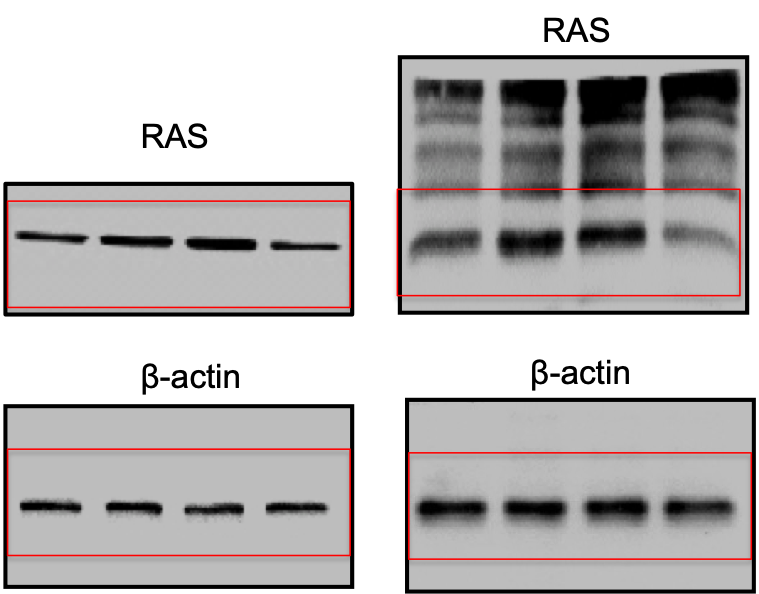


Multiple exposures


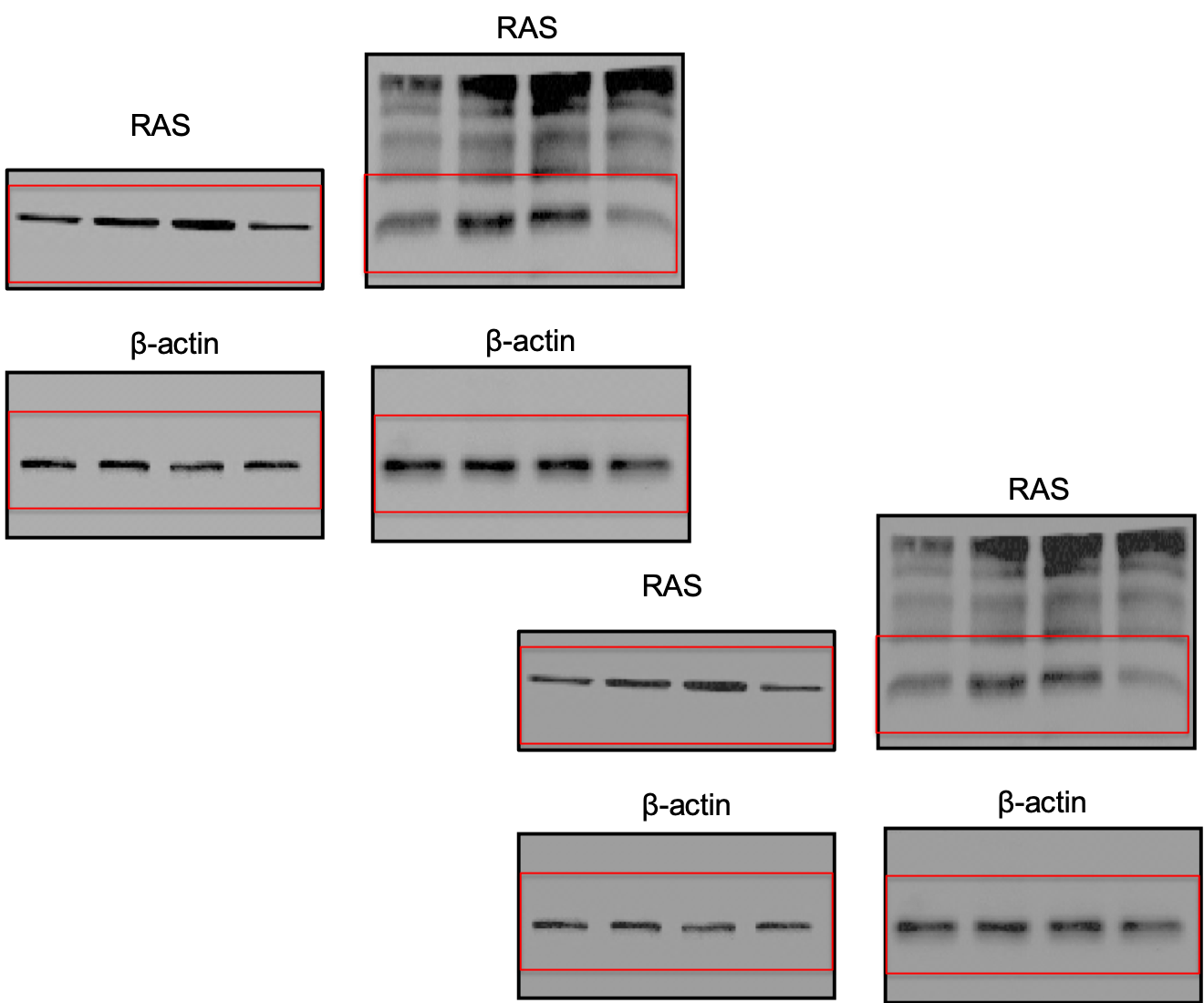


Full-length gels and blots for figure 4E and F.


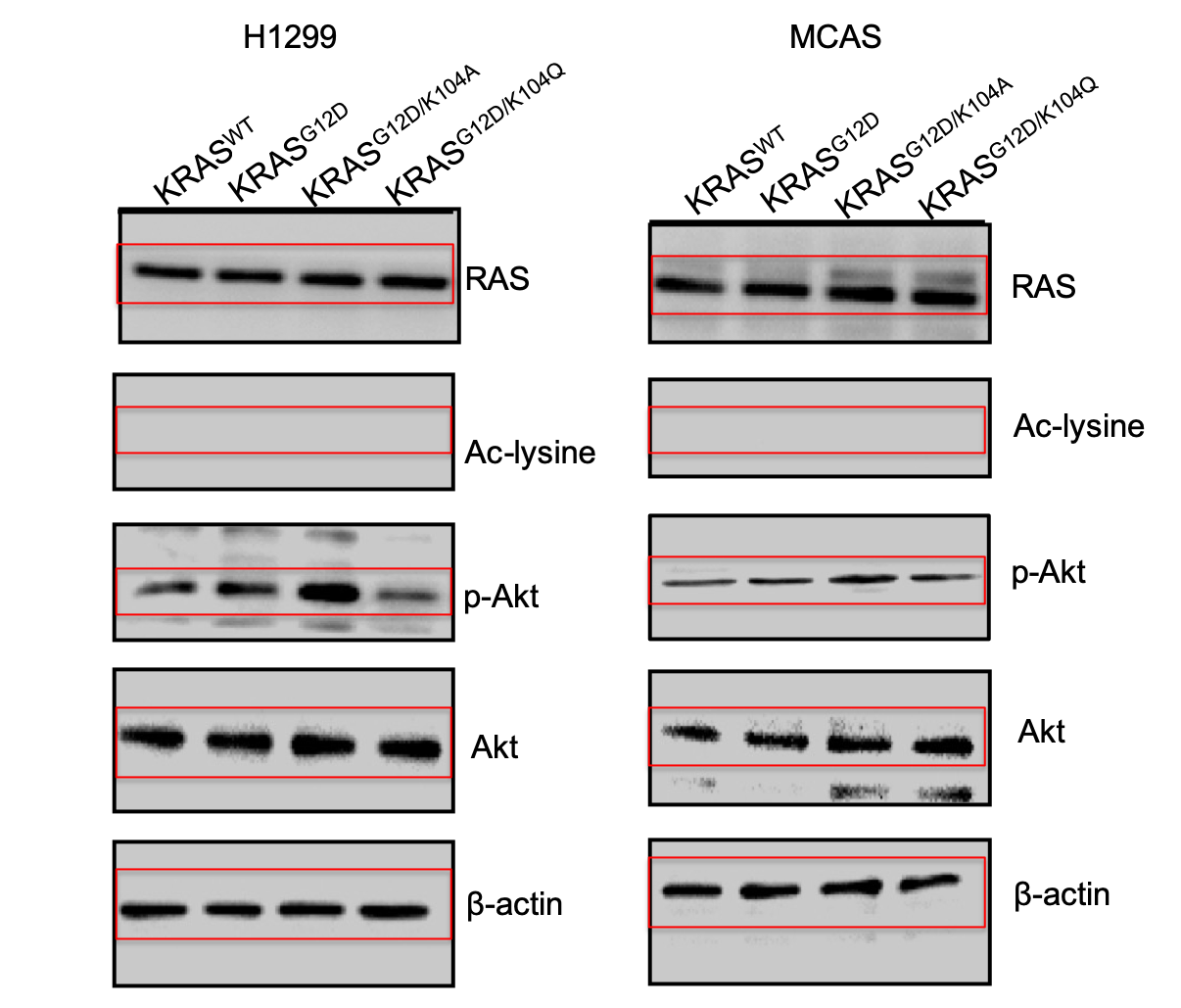


Multiple exposures


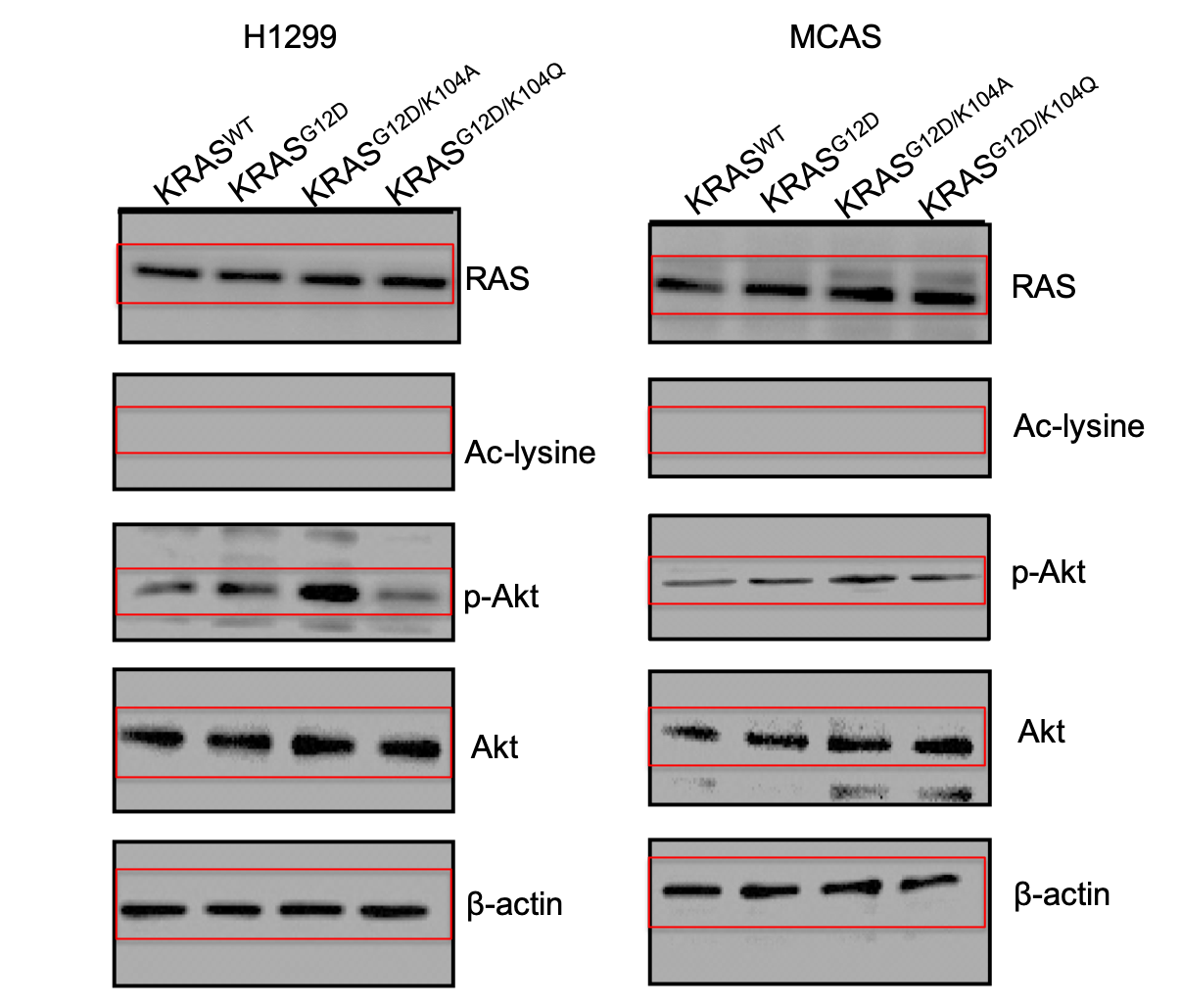

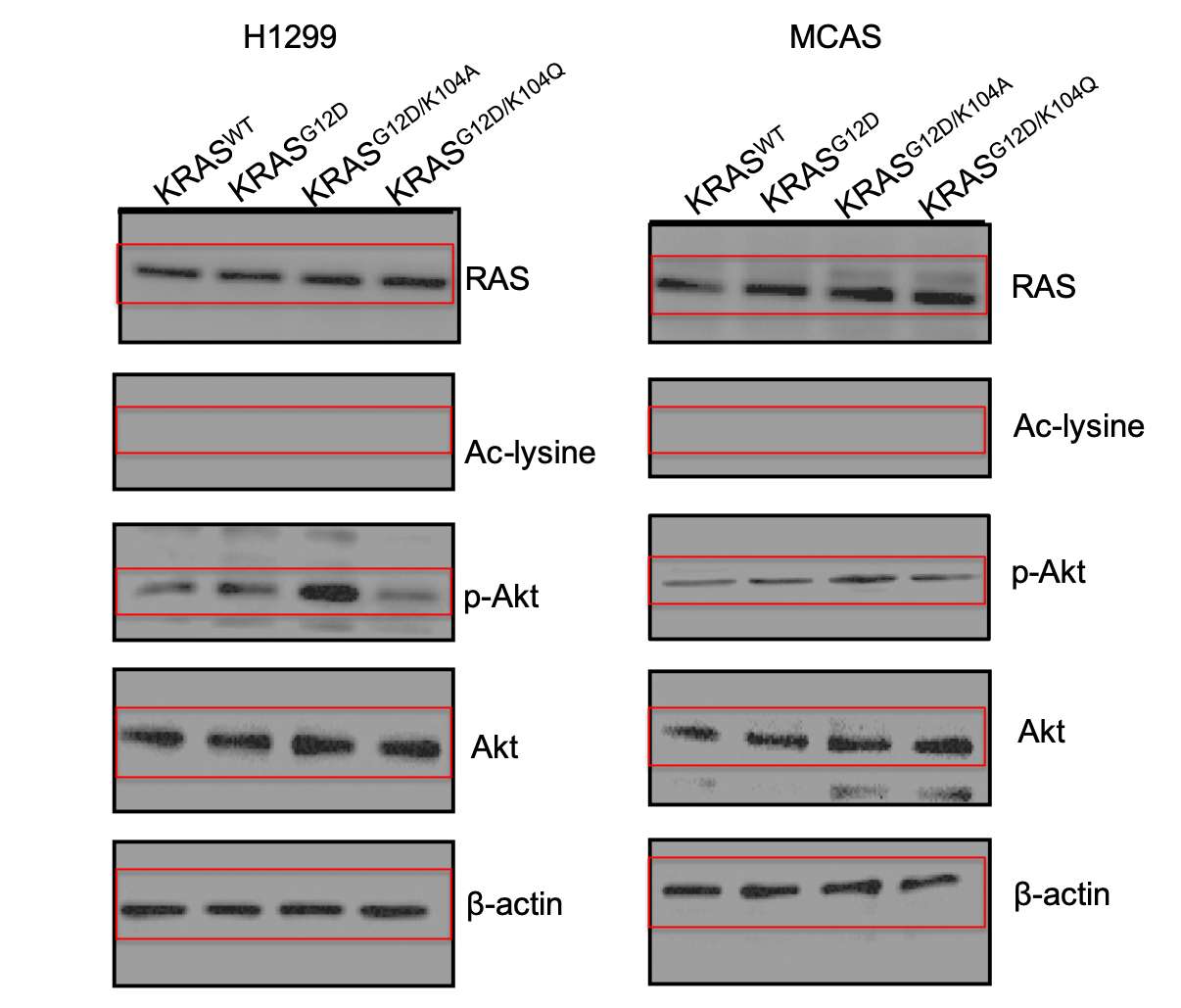

Supplement: Supplementary file 1 — Supplementary file1. [file 41598_2020_74463_MOESM1_ESM.docx]
